# Dome‐Patterned Metamaterial Sheets

**DOI:** 10.1002/advs.202001955

**Published:** 2020-10-07

**Authors:** Jakob A. Faber, Janav P. Udani, Katherine S. Riley, André R. Studart, Andres F. Arrieta

**Affiliations:** ^1^ School of Mechanical Engineering Purdue University 585 Purdue Mall West Lafayette IN 47907 USA; ^2^ Department of Materials Complex Materials ETH Zürich Vladimir‐Prelog‐Weg 5 Zürich 8093 Switzerland

**Keywords:** hierarchical multistability, mechanical metamaterials, mechanologic, soft robotics

## Abstract

The properties of conventional materials result from the arrangement of and the interaction between atoms at the nanoscale. Metamaterials have shifted this paradigm by offering property control through structural design at the mesoscale, thus broadening the design space beyond the limits of traditional materials. A family of mechanical metamaterials consisting of soft sheets featuring a patterned array of reconfigurable bistable domes is reported here. The domes in this metamaterial architecture can be reversibly inverted at the local scale to generate programmable multistable shapes and tunable mechanical responses at the global scale. By 3D printing a robotic gripper with energy‐storing skin and a structure that can memorize and compute spatially‐distributed mechanical signals, it is shown that these metamaterials are an attractive platform for novel mechanologic concepts and open new design opportunities for structures used in robotics, architecture, and biomedical applications.

## Graphical abstract

A dome‐patterned mechanical metamaterial displaying extraordinary property adaptation, shape‐shifting, and intrinsic mechanologic is presented. This displays an exponentially large and unique set of programmed global states that can be accessed by specific spatiotemporal deformations. This programmability is applied to designing soft robotic grippers that actuate without external energy sources and the collocation of memory and computation as a material property.

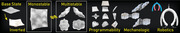

## 1 Introduction

Mechanical metamaterials exploit geometrical arrangements to manipulate the deflections, stresses, and strain energy of building blocks to generate unconventional mechanical properties at the larger macroscale. Prominent examples of metamaterials are auxetic structures that expand laterally when stretched,^[^ ^1^, ^2^ ^]^ origami‐ and kirigami‐inspired sheets that are foldable in multiple global geometries,^[^ ^3^, ^4^, ^5^, ^6^, ^7^ ^]^ and architected periodic lattices that rely on size effects to achieve superior strength with minimal weight.^[^ ^8^ ^]^ In addition to decoupling global mechanical response from intrinsic material properties, mechanical metamaterials also feature large‐area reconfiguration, adaptive, and morphing capabilities that are unmet by conventional materials.^[^ ^9^, ^10^, ^11^ ^]^ Such functionalities are often achieved by incorporating bistable elements into the unit cells of the metamaterial. Buckling beams^[^ ^12^, ^13^, ^14^ ^]^ or bending plates^[^ ^15^ ^]^ are typical examples of bistable motifs. By providing local energy minima in the structural configuration space, such bistable elements allow metamaterials to carry mechanical loads, locally store elastic energy in the structure and/or generate multiple stable reconfigurable geometries.^[^ ^16^, ^17^ ^]^ These functionalities have been harnessed to design deployable structures,^[^ ^18^, ^19^ ^]^ impact absorbers,^[^ ^20^, ^21^, ^22^ ^]^ robotic actuators,^[^ ^23^, ^24^ ^]^ energy harvesting,^[^ ^25^, ^26^ ^]^ and micromechanical systems,^[^ ^27^, ^28^ ^]^ waveguiding systems,^[^ ^29^, ^30^, ^31^ ^]^ memory^[^ ^32^, ^33^ ^]^ and logic devices,^[^ ^34^, ^35^, ^36^, ^37^ ^]^ and morphing elements in architecture. In particular, multistability in metamaterials allows for programming both static and dynamic properties, such as stiffness adaptation,^[^ ^6^, ^38^ ^]^ tunable bandgaps,^[^ ^39^, ^40^ ^]^ and quantum valley Hall effect.^[^ ^41^ ^]^

The ability to generate multiple stable states is particularly compelling since it allows for reversible morphing and memory effects that can be directly programmed in the metamaterial architecture. Inspired by the multistable states of crumpled sheets, a metamaterial architecture with vertices interconnected by springs has been proposed to create reconfigurable complex shapes. This is based purely on geometry instead of plastic deformation of the constituent material.^[^ ^42^ ^]^ The unparalleled functionalities demonstrated so far have led to a rich library of metamaterial designs that continues to expand. The physical realization of such designs has now opened opportunities to translate multimaterial concepts to a wider range of functional applications.

The translation of metamaterial designs into functional applications calls for the development of geometries that are simple enough to be fabricated with a broad range of material chemistries, but also sufficiently complex to enable the implementation of bistable behavior. Domes offer a simple geometry that can exhibit bistable behavior and be easily manufactured in mechanically‐robust sheets without damage‐prone sharp edges and vertices. For a given set of geometric boundary conditions, the domes' energy landscape has been shown to depend only on the dome height, the sheet thickness, and the Poisson's ratio of the constituent material.^[^ ^43^, ^44^ ^]^ Domes may be patterned to further increase their potential to realize shape adaptation.^[^ ^45^ ^]^ Previous work has also revealed that structures featuring a periodic array of domes can be fabricated from plastics or by stamping metal sheets at high temperatures.^[^ ^46^, ^47^ ^]^ However, the metamaterial features of these structures have not yet been fully explored, with attention concentrated on the auxetic behavior,^[^ ^48^, ^49^ ^]^ surface‐fluid interactions,^[^ ^50^ ^]^ and optical properties from local morphing.^[^ ^51^ ^]^ Allowing the constituent material to be softer should enable reversible inversion of domes and full re‐programmability of the metamaterial sheet. Dome‐patterned soft sheets may also be seamlessly integrated into functional objects, unraveling a range of opportunities in applications from robotics to medical technologies to architecture.

**Figure 1:**
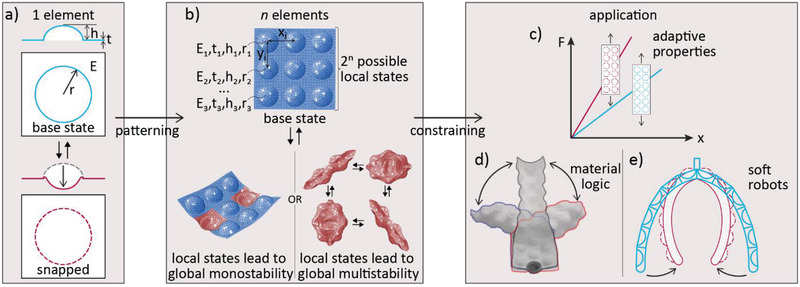
Design of dome‐patterned metamaterial sheets featuring complex shapes, memory effects, programmable mechanical response, and muscle‐free actuation. a) Schematics of an individual dome in two possible stable states: the base state as manufactured (blue) and an inverted, pre‐stressed state (red). The elastic modulus of the sheet material, sheet thickness, dome height, and dome radius are indicated by *E*, *t*, *h*, and *r*, respectively. b) Combining individual domes into large‐area patterns yields metamaterial sheets with global multistable shapes and mechanical response that can be programmed through material selection and local geometry. c) Global properties can be tuned in unintuitive ways by local state reconfiguration. d) Material computation can be encoded by profiting from the history‐dependence of the dome inversion pattern to reach a particular global shape, three of which are shown. This radically expands the response space, making these sheets ideal candidates for computing and logic through deformation. e) Functional devices can be created by constraining the wide number of possible configurations into selected geometries and patterns. This is schematically illustrated by a soft robotic actuator that changes its global equilibrium shape through air‐driven inversion of local domes.

We investigate this rich metamaterial system with the help of experiments and simulations performed at both the local and global length scales. First, the energy landscape associated with the local inversion phenomenon is simulated to define the geometrical parameters and material properties required to manufacture individual bistable domes (**Figure** **1**a). Next, strips and sheets displaying an array of locally addressable bistable domes are numerically and experimentally studied in terms of global shape and multistability (Figure 1b, top). Importantly, we demonstrate a hierarchical multistability behavior that shows coexisting global solutions for a single pattern of inverted domes (Figure 1b, bottom). The different families of coexisting global shapes that emerge are then classified to demonstrate the rich shape programmability of the considered sheets. Finally, the proposed dome‐patterned metamaterial design is harnessed to (i) manufacture sheet materials with adaptive global properties depending on their local states (Figure 1c); (ii) utilize the path dependency of our metamaterial to demonstrate that coexisting global shapes can encode deformation history, which could be utilized to conduct logic operations for mechanical computation^[^ ^52^, ^53^, ^54^ ^]^ (Figure 1d); and (iii) create a two‐finger gripper that can exert macroscopic grabbing forces even in the absence of external energy input, such as pressurization or muscular action (Figure 1e).

## 2 Energetics: Global Deflection of 1D Strips

The energetics involved during inversion of an individual dome were investigated through finite element (FE) simulations and experiments on 3D printed soft domes with controlled height and thickness (Figure S1a,b, Supporting Information). Bistability was found to strongly depend on geometrical form factors of the dome in agreement with previous works.^[^ ^55^, ^56^, ^57^, ^58^ ^]^ This provides a route to design the energy landscape of the system that is not restricted by the material choice. Our experiments show that a minimum dome height (*h*_min_) is required to achieve bistability. Such minimum height was found to scale linearly with the thickness of the dome (*t*) (Figure S1c, Supporting Information).

**Figure 2:**
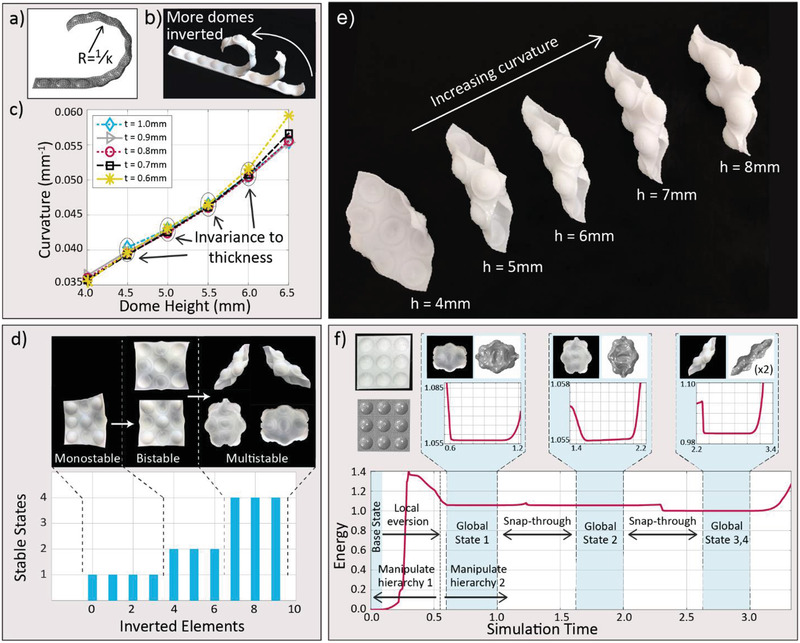
Shape changes and hierarchical multistability of dome‐patterned strips and sheets. a) FE simulation of a dome‐patterned strip, illustrating the global curvature *K* generated upon inversion of selected domes. b) Superimposed photographs of a 3D printed dome‐patterned strip with an increasing number of inverted domes. c) The effect of the dome height and strip thickness on the simulated global curvature of strips after inversion of all domes. d) The number and associated photographs of experimentally observed multistable states as a function of the number of inverted domes in a 3 × 3 array. e) Global curvature of patterned sheets featuring a 3 × 3 array of domes of different heights, *h*. f) Simulated energy landscape of a hierarchically multistable 3 × 3 sheet. The manipulation of local elements activates additional stable shapes on the global level. Energy units are nondimensionalized as the ratio of energy to the lowest global stable state energy with inverted domes, which is the twisted state in this case.

Once the geometrical conditions required to obtain bistable domes are fulfilled, programmable metamaterials can be generated by combining multiple domes in the form of strips or sheets (**Figure** **2**). Inverting individual domes of the resulting strips and sheets leads to global changes in the shape and mechanical response of the dome‐patterned metamaterial at the next hierarchical level. To enable programmability of such systems, we study the effect of the geometry of the domes on the global curvature of exemplary strips (Figure 2a,b). For sufficiently small dome heights (*h* < 6 mm for *r* = 8 mm), the experiments indicate a linear dependence of the global strip curvature on the dome height. This linear scaling is effectively quantified by FE simulations (Figure 2c and Figure S2, Supporting Information). Interestingly, the global curvature of the strip does not depend on the dome thickness if the dome height is kept below 6 mm. To understand the physics underlying this effect and establish an analytical correlation between the local dome inversion and the global curvature of the dome‐patterned strips, we propose a simple mechanical model that considers the energies involved at the two distinct length scales. Our analysis reveals that a dome in a strip is elastically constrained by the surrounding flat region of the strip and therefore requires more work to invert than an unconstrained dome. This work differential is stored as pre‐stress, which results in the bending of the strips (Figures S3, Supporting Information). This model effectively captures the linear dependence of the strip curvature on the height of the dome and explains why the global geometry is not affected by the strip thickness.

## 3 Hierarchical Multistability in 2D Metasheets

The 1D array of domes in the strip can be extended to 2D patterns to create metamaterial sheets with rich multistable behavior (Figure 2d). Taking a sheet with a thermoplastic polyurethane (TPU) 3 × 3 array of domes as an example, we observe that the number of possible coexisting stable states on the global level increases with the number of locally inverted domes. In this example, up to four possible global stable states can be generated if all the domes are inverted. Two of these possible states correspond to a twisting geometry, the curvature of which can be incremented by increasing the height of the individual domes (Figure 2e). This direct correlation between dome height and global curvature resembles that observed for the simpler strip geometry. We use FE simulations considering a linear elastic material model and geometric nonlinearity to determine the energy landscape of the 3 × 3 metamaterial architecture at multiple length scales (Figure 2f). Interestingly, simulations of this particular system show that the twisted states exhibit a lower strain energy level than the cylindrical shapes, indicating greater stability for the former. In addition, the energy barriers dividing the coexisting stable states at the global hierarchical level are considerably smaller than the energy barriers to invert individual domes at the local length scale. This facilitates transitions between different global shapes, while preserving the configuration of inverted elements programmed at the dome level. Sheets with all domes inverted represent just one of the many possible configuration states that can be programmed in the metamaterial. By manipulating the number and distribution of inverted elements in 3D printed sheets, distinct monostable, bistable, and multistable states can be generated in a simple 3 × 3 square array of domes (Figure 2d). Importantly, material viscoelasticity plays no major role in the hierarchical multistable behavior of our metasheets. We illustrate this by manufacturing 3 × 3 arrays with a less viscoelastic material nylon (Figure S10, Supporting Information), which displays similar qualitative behavior to that obtained with the TPU metasheets.

**Figure 3:**
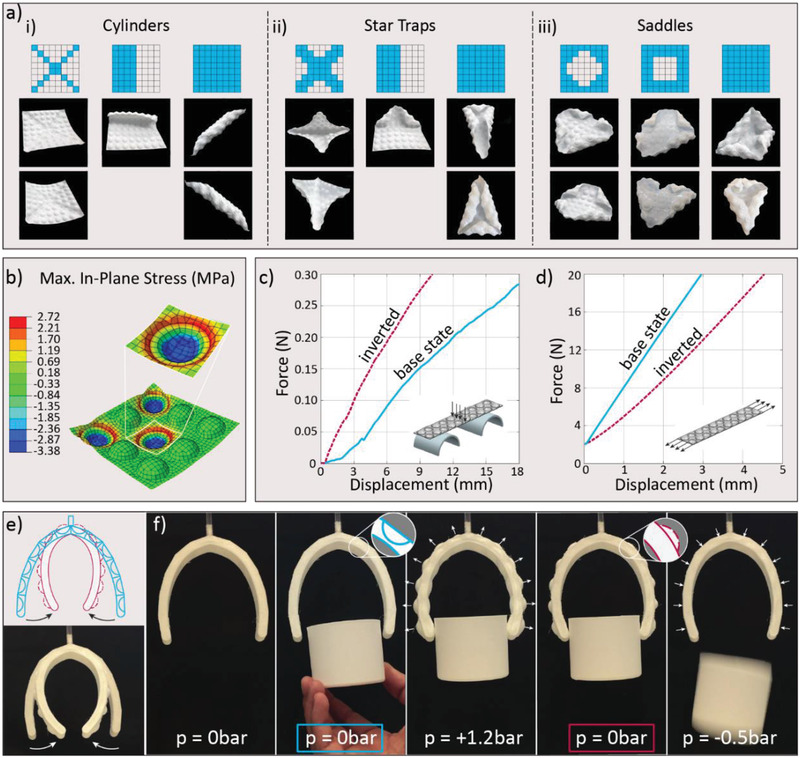
Complex multistable shapes, programmable mechanical response, and muscle‐free actuation enabled by dome‐patterned metamaterials. a) Examples of complex multistable shapes that can be generated using 8 × 8 sheets with specific patterns of inverted domes with normalized Gaussian curvatures of 0, 1, and −1, associated with (i) cylinders, (ii) doubly curved stars, and (iii) saddles, respectively. b) FE analysis of individual domes in a 3 × 3 sheet illustrating the stresses developed upon inversion. c,d) Force‐displacement relations for dome‐patterned sheets subjected to bending (left) and stretching (right). Sheets with an alternating arrangement of inverted domes are compared with a reference sample without dome inversion. The alternating arrangement of inverted domes was used because the induced up‐ and down‐curvatures equalize each other, preventing changes in the global shape of the sheet. e) Pneumatic robotic gripper displaying a dome‐patterned outer skin. f) Snapshots of the gripper at different applied pressures illustrate the reversible inversion of domes and how this can be exploited to exert a grabbing force on an object, even in the absence of pressurization.

Increasing the number of invertible domes in the sheet leads to an explosion of the number of possible configurations at the global scale. In contrast to metamaterial architectures based on buckling and bending elements, no straightforward correlation exists between the local state of the bistable unit and the global geometry of the entire structure. Breaking this relationship opens a new avenue towards substantially larger reconfigurability, allowing for the creation of several overall global geometries depending on the chosen pattern of inverted domes or, in other words, the layout of local states. To illustrate the remarkable level of shape complexity and multistable states achievable with these hierarchical metamaterials, we 3D printed exemplary sheets of 8 × 8 square arrays of domes (Figure S4, Supporting Information). Indeed, a rich library of complex, coexisting shapes emerges at the global scale by programming the pattern of locally inverted domes (**Figure** **3**a). Importantly, the global shape generated by one particular pattern of inverted domes can be shifted between different multistable geometries upon gentle manipulation of the sheet. This reflects the low energy barrier between multiple global states and the deep energy well that keeps the domes locally inverted. Analogously, the energy barrier between global shapes can be widened, if desired, by increasing the sheet's thickness and deepening the domes, highlighting the versatility of the approach.

Despite the non‐trivial relation between local and global states, exemplary families of multistable geometries can be generated by programming the pattern of inverted domes into specific arrays (Figure 3a and Figure S5, Supporting Information). We illustrate the wide variety of possible multistable structures by organizing families of surfaces in terms of their normalized Gaussian curvatures (*K*). Structures with *K* values of 0, +1, and −1 are showcased using cylindrical, star trap, and saddle shapes, respectively. These geometries can be attained inputting distinct patterns of inverted domes. Inverted domes forming a cross pattern, for instance, lead to slightly curved cylindrical geometries or star‐like shapes with highly curved edges, depending on the pattern of domes inverted. Limiting the inverted domes to only one half of the squared sheet allows one to induce positive (*K* = +1) or zero (*K* = 0) Gaussian curvatures in specific areas of the metamaterial. Sheets with site‐specific curvatures are also achievable using other non‐centrosymmetric patterns (Figure S5, Supporting Information). In another example, saddle‐like shapes (*K* = −1) can be obtained if only domes close to the edges of the sheet are inverted. Finally, the inversion of all the domes in the sheet results in highly curved surfaces with multiple coexisting stable shapes covering all three geometrical families. This method of transforming flat (2D) sheets into curved (3D) surfaces provides an alternative mechanism to origami for attaining complex global shapes. In contrast to origami, our patterned metamaterials display intrinsic stiffness, thus naturally yielding a general method to obtain load‐carrying, morphing structures.

## 4 Mechanical and Robotic Materials Programmability

Besides hierarchical multistable shapes, the local inversion of domes also enables tuning of the global mechanical behavior of the metamaterial sheet. This effect arises from the high elastic energy stored along the edge of the inverted dome, which introduces localized pre‐stressed areas and out‐of‐plane deflections into the surrounding sheet material. FE simulations of a 3 × 3 sheet with inverted domes show the compressive and tensile stresses developed on the flat boundary regions, as well as its deformed topography (Figure 3b and Figure S6, Supporting Information). We quantify the effect of this pre‐straining on the global mechanical response of the metamaterial by measuring the force‐displacement relation of sheets with a specific inversion pattern when subjected to bending (Figure 3c) and stretching (Figure 3d).

The experimental results show that the inversion of domes in this specific pattern changes the stiffness of the sheet by twofold. Interestingly, opposite trends are observed for each one of the investigated mechanical loading modes. Whereas dome inversion increases the stiffness of the sheet under bending, a softening effect is achieved if the sheet is probed under tension (Figure 3b,c). These results can be rationalized by analyzing the interplay between the elastically stored local pre‐stresses, the local deformation modes activated by inversions, and the remotely applied stretching and bending loads. While some stress–strain nonlinearity is present and can lead to changed stiffness due to pre‐stress, the geometrical reconfiguration dominates the property changes. In the base state, the areas in between domes are flat and, therefore, compliant in bending. When distorted by surrounding inverted elements, these areas become profiled. This is equivalent to an increase in the bending stiffness of metal sheets by the introduction of dimples, which increase the second moment of inertia.^[^ ^47^ ^]^ In contrast, when subject to tensile stress, the profiled areas of the inverted state act as undulations in the direction of load. These do not form a direct load path in the tensile direction, reducing the sheet's stiffness. Only as the waviness is straightened out at higher loads, the metamaterial experiences a more equally stressed state and its tensile modulus approaches the base state value. As discussed before, the programmability of our metasheets is driven by purely geometrical effects. We illustrate this by conducting tensile FE simulations of a sheet using polylactic acid as the constitutive material (Figure S11, Supporting Information). We observe similar property programmability as obtained with TPU, while the absolute force values increase commensurably with the higher material modulus. Furthermore, our simulations indicate that the sheets can be designed to exhibit strains under 2% when all the domes are inverted and during loading events. These strains allow for using high‐modulus engineering materials such as hybrid composites^[^ ^59^ ^]^ capable of withstanding strains of up to 2%.

Overall, our results demonstrate the possibility of programming the global mechanical response of the metamaterial sheet through the local inversion of individual domes. By changing the local geometry of the domes, the intrinsic properties of the constituent material, and the dome inversion pattern, it is possible to design reconfigurable metamaterial sheets featuring a broad range of programmable global mechanical properties. The dominant effect of local geometry highlights the possibility to attain the demonstrated multistable behavior and mechanical programmability independently of the constitutive material used for the sheet. This provides a general route to obtain re‐programmable hierarchical metamaterials.

The programmable shape‐shifting and global mechanical response of the metamaterial sheet can be harnessed to build specific geometries that are able to perform targeted functions. We illustrate this concept by designing and 3D printing a muscle‐free pneumatic‐driven gripper for soft robotic actuation (Figure 3e and Movie S1, Supporting Information). The gripper features two finger‐like chambers that can be pressurized through an inlet connected to an external pump. The outer surface of each finger displays a single‐row array of concave domes with geometry and intrinsic material properties that allow for reversible inversion. Domes are pneumatically inverted on‐demand to programmed configurations in response to a single input and without feedback control by selectively pressurizing the chamber (Movie S2, Supporting Information). The tests are repeatable, and viscoelasticity is found to have no effect on the final shape, other than a minor delay in the time needed to achieve the final shape (Movie S3, Supporting Information). A similar pressurization concept was previously used for the actuation of origami‐inspired elastomeric, structurally bistable units.^[^ ^60^ ^]^ Experiments show that the distance between the fingertips can be reduced up to 79%, from 92 to 19 mm upon pressurization with 1.2 bar (Figure 3e). Most importantly, the local inversion of the domes leads to a global change in the gripper's equilibrium shape when the pressure is removed. As a result, gripping forces can still be applied even if the finger‐like chambers are fully depressurized (Figure 3f). This feature arises from the strain energy stored in the dome‐patterned metamaterial sheet and cannot be achieved using conventional actuators. A similar so‐called “catch” functionality is observed in smooth muscular tissue, where biomolecules are presumably used to minimize the energy needed to maintain actuation.^[^ ^61^ ^]^ More generally, the strain energy stored in the metamaterial as pre‐stress enables this inherent gripping ability, in which the structure itself serves as one of the elements in an agonistic‐antagonistic pair. This principle is used in nature by insects to simplify actuation systems.^[^ ^62^ ^]^ Despite the very different materials, chemistries, and length scales involved, the individual domes of our soft actuator can also be conceptually viewed as a mechanical analog of the sarcomere units present in the muscular system of vertebrates.^[^ ^63^, ^64^ ^]^ As in these biological muscles, the mechanism of action of our soft actuator relies on a modular linear architecture capable of generating large deformations through the local relative lengthening enabled by individual domes.

## 5 Morphologic Computation with 2D Metasheets

**Figure 4:**
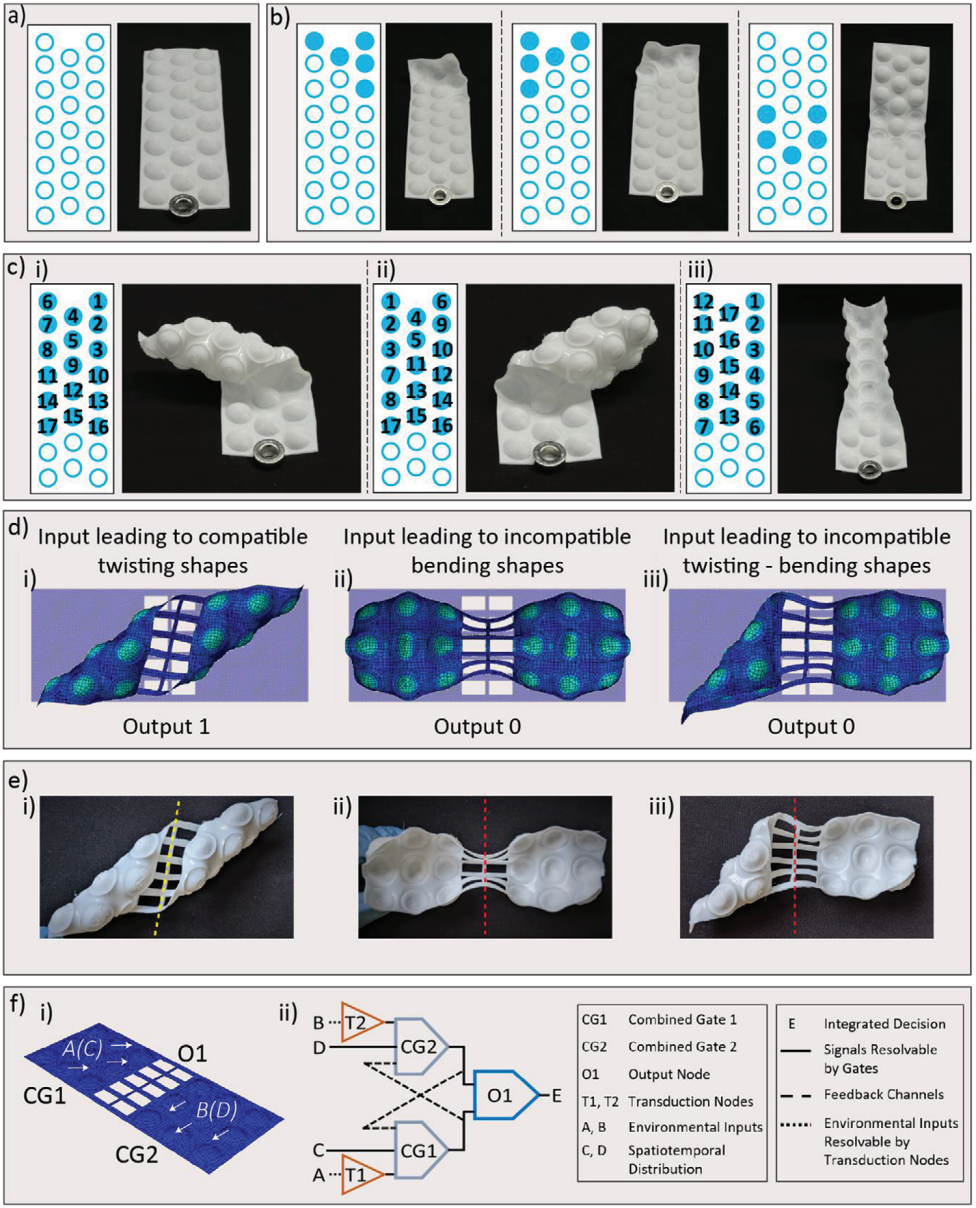
Memory and morphology of dome‐patterned metamaterials. a–c) The correlation between the final shape and the inversion pattern and history allows for storing mechanical information and conducting in‐memory logic operations based on the deformations imposed on our sheets. (a) Zero stress state. (b) Different inversion patterns leading to single global shapes independently of the followed order (Movie S4, Supporting Information). (c) Three different inversion histories reaching the same final inverted dome pattern but distinct global shapes: twist‐left (Figure 4c.i, Figure S7b, and Movie S5, Supporting Information), twist‐right (Figure 4c.ii, Figure S7c, and Movie S6, Supporting Information) and cylindrical (Figure 4c.iii, Figure S7d, and Movie S7, Supporting Information). d) Simulations depicting morphologic operations using two connected metasheets (Figure 4d): (d.i) input field leading to twisting shapes for the two metasheets (Movie S8, Supporting Information), (d.ii) input field leading to bending shapes (Movie S9, Supporting Information), and (d.iii) input fields leading to a twisting‐bending shape (Movie S10, Supporting Information). The history and pattern of inverted domes induce rotation of the central, dashed yellow line when the lowest strain energy global state is reached, interpreted as Output 1 (Figure S8f, Supporting Information). No rotation indicates that a coexisting, higher strain energy state is reached, interpreted as Output 0 (dashed red line Figure S8f, Supporting Information). e) Experimental global shapes matching Output 1 (e.i) and Output 0 (e.ii‐iii). f) The spatiotemporal mechanical input pattern (white arrows), collective deformation, and state of center line (output node O1) can be utilized to establish a state transition table (Figure S9, Supporting Information) to provide a direct decision (signal E) in response to spatially distributed environmental inputs, A(C) and B(D), causing deformation of the two connected metasheets. An abstract logic diagram is shown in (f.ii): input patterns on metasheets (CG1 and CG2) lead to bending or twisting shapes of the metasheets (bending, Figure S8b, Supporting Information, and twisting, Figure S8c, Supporting Information), the compatibility of which yields an integral signal represented by Output E.

Unique to our metamaterial architecture, the attained global shape may depend not only on the pattern but also on the history of local inversions on the metasheet (**Figure** **4** a–c). Inversion patterns with few inverted domes with respect to the total number on the sheet can lead to single global shapes independently from the history (Figure 4b and Movie S4, Supporting Information). In contrast, denser patterns show history‐dependent morphing capabilities that allow us to arrive at specific global shapes from the available multiple coexisting states (Figure 4c, Figure S7 and Movies S5–S7, Supporting Information). Importantly, the correlation between inversion history and global shape allows us to encode memory and path‐dependent logics into the material architecture. This opens the possibility to expand current mechanologic concepts,^[^ ^36^, ^37^, ^52^ ^]^ from transistor‐based logic to more complex computation paradigms. We introduce the term “morphologic metamaterials” to refer to metamaterial sheets that can perform logical operations and store information using changes in shape, as opposed to the changes in electrical potential and flow in conventional electronic materials.

We demonstrate the mechanical memory and computation capabilities of our morphologic metamaterial by using its hierarchical multistable behavior to: a) store information in binary form by setting an individual unit cell to the ground state as 0 or inverted state as 1; and b) perform logic operations by ascribing coexisting global shapes to both the spatial distribution and the sequence of inputs. Spatial distribution refers to which domes have been inverted, whereas the sequence of inputs corresponds to the order in which domes have been inverted (Figure 4c and Movies S8–S10, Supporting Information). The memory effect is readily manifested by the dependence of the final global shape on the history of dome inversion. Such history‐dependence allows us to encode the sequence of input inversion events into the final hierarchical state adopted by our dome‐patterned metamaterial. As a result, the local unit cell states store memory of the domes that were inverted, whereas the final shape stores the history of inversion.

The ability to perform logic operations is shown by exploiting the compatibility between the global shapes of two sheets that are elastically connected by beam‐like links (Figure 4d, Figures S8 and S9, Supporting Information). The links establish a common constraint between the sheets and are used as the readout of the computation. If the history of dome inversion imposed on the two sheets is such that their global states do not naturally lead to the twisted lower energy level (Figures 4d.ii–iii), the deformations are incompatible, and the readout remains straight yielding 0 as output. Conversely, if the history of inversion on the two sheets is compatible, a coherent final shape is reached, resulting in a slanted readout yielding 1 as output (Figure 4d.i). This deformation compatibility can thus be interpreted as a logic abstraction that yields not only the inversion pattern, but also uniquely the history of the mechanical inputs to the sheets. Previous examples of mechanologic have relied on bookkeeping on an external device, thus separating the computation unit from the memory unit. In contrast, our morphologic metamaterials depart from von Neumann‐like architectures for mechanical computation by showing a type of in‐memory material mechanologic akin to neuromorphic computation architectures.^[^ ^65^, ^66^ ^]^ Moreover, latches in conventional computational architectures are able to store one memory state, while our metamaterial architecture can resolve many intermediate states based on the input field and drive computations. This enables in‐situ decision‐making capabilities based on the mechanical interactions with the environment and the associated unique hierarchical multistable behavior.

## 6 Conclusions

In summary, dome‐patterned sheets can be designed to display an extraordinary range of mechanical properties, shaping capabilities, and in‐memory mechanologic effects that are reversibly programmed within the metamaterial architecture. We show the characteristics of our sheets are determined by the geometry (architecture) and not by specific constitutive material properties. In contrast to other multistable metamaterial architectures with equal units, our metamaterials display distinct spatiotemporal deformation sequences allowing to access specific global states. This allows us to circumvent the difficulty in predicting the final states in equipotential multistable metamaterials^[^ ^67^ ^]^ without recourse to imperfections or variations of unit cell properties. Thus, our metamaterial architecture provides a vast design space for the deterministic realization of structures covering a spectrum of properties and functionalities far beyond those accessible using conventional materials. In analogy to atomic mechanisms, the global shape and mechanical properties of the metamaterial are tuned by storing elastic strain energy and introducing finite local deformation in the underlying structure. Importantly, our sheets extend beyond atomic mechanisms by providing coexisting global behaviors for a single constitutive pattern, thereby opening exponentially large possibilities for property design. Because these local effects can be reversibly programmed through the inversion of individual domes, the energy landscape of the proposed metamaterial sheets is fully programmable and can be tailored to fulfill specific functionalities. The physical realization of a robotic gripper that sustains an actuation action even in the absence of external forces is just one of the many functionalities envisioned for this new class of mechanical metamaterials. We also demonstrate mechanically‐driven collocation of memory and computation exploiting the nonlinear relationship between local inverted domes and global shapes. We refer to material systems displaying this behavior as morphologic metamaterials. This capacity can be viewed as an intrinsic material property of our sheets combining into a mechanical metamaterial multiple functions previously demonstrated separately, such as distributed sensing,^[^ ^53^ ^]^ mechanical memory,^[^ ^32^ ^]^ and morphological,^[^ ^68^, ^69^ ^]^ and reservoir computing.^[^ ^70^ ^]^ The highly coupled morphing and history‐dependent inherent morphologic propertyies of our sheets are ideal for designing octopus‐inspired^[^ ^71^ ^]^ peripheral computation centers for flexible structures and soft robotics. These can be programmed to realize reflexive‐type responses, emulating short neural circuits in organisms in which the sensing, computation, and reconfiguration are integrated into a single arrangement. The morphological information processing of our sheets departs from binary‐based logic and serves as a blueprint for metamaterials that compute as physical reservoir computers.^[^ ^72^ ^]^

## Author Contributions

All authors designed the research. K.S.R. and J.A.F. conducted single dome characterization FE and experiments. J.P.U., J.A.F., A.F.A., and A.R.S. developed the mechanics model. J.P.U. designed dome strip curvature and energetics FE simulations. J.A.F. designed hierarchical multistability FE simulations. J.A.F., J.P.U., and K.S.R. conducted hierarchical mutistability experiments. J.A.F. conducted mechanical load response experiments and robotic gripper experiments. J.P.U. and A.F.A. designed and developed the memory and morphologic features. J.P.U. conducted the morphologic and programmability FE simulations and experiments. All authors contributed to the writing and revision of the manuscript and supporting information at different stages, and A.R.S. and A.F.A. provided guidance throughout the research.

## Conflict of Interest

The authors declare no conflict of interest.

## Supporting information

Supporting Information

Supplemental Movie 1

Supplemental Movie 2

Supplemental Movie 3

Supplemental Movie 4

Supplemental Movie 5

Supplemental Movie 6

Supplemental Movie 7

Supplemental Movie 8

Supplemental Movie 9

Supplemental Movie 10
