# Supplementary material for: Dome‐Patterned Metamaterial Sheets
Source: Adv Sci (Weinh). 2020 Oct 7;7(22):2001955. doi: 10.1002/advs.202001955 (PMC7675196; doi:10.1002/advs.202001955)
Supplement: Supplementary file 1 — Supporting Information [file ADVS-7-2001955-s001.pdf]

WILEY-VCH

© 2020 Wiley-VCH GmbH

ADVANCED SCIENCE
Open Access

Supporting Information

for *Adv. Sci.,* DOI: 10.1002/advs.202001955

Dome-Patterned Metamaterial Sheets

Jakob A. Faber, Janav P. Udani, Katherine S. Riley, André R. Studart, and Andres F. Arrieta

© 2020 Wiley-VCH GmbH

Supporting Information

Dome-Patterned Metamaterial Sheets

Jakob A. Faber,[1,2] Janav P. Udani,[1] Katherine S. Riley,[1] André R. Studart[2], Andres F. Arrieta[1*]*

[*]*Corresponding authors*

Dr. J. A. Faber, J. P. Udani, K. S. Riley, Prof. A. F. Arrieta

[1]School of Mechanical Engineering and Ray W. Herrick Laboratories, Purdue University, 585 Purdue Mall, West Lafayette, IN, USA

E-mail: aarrieta@purdue.edu

Dr. J. A. Faber, Prof. A. R. Studart

[2]Department of Materials, Complex Materials, ETH Zürich, Vladimir-Prelog-Weg 5, 8093 Zürich, Switzerland

E-mail: andre.studart@mat.ethz.ch

This file includes:

1. Materials and Methods
2. Mechanics Model
3. Surface family selection from multistable metasheets
4. Localized areas of pre-stress for property tunability
5. Soft robotic structures with programmed minimum-energy states
6. Morphologic computation with multistable metasheets
7. Role of constituent material modulus on hierarchical multistability and property programmability
8. Figures S1 – S11
9. List of Movies S1 – S10

10. References

1. Materials and Methods

Single dome characterization - Experiments

Single domes were modeled using SolidWorks 2015. Uniform thicknesses ranging from 0.5 to 1.4 mm in steps of 0.1 mm and dome heights ranging from 2 to 8 mm in steps of 1 mm were combined in a full factorial design. The dome diameter was kept constant at 16 mm and the outer sheet dimensions were 20 mm × 20 mm. The 3D files were sliced with Ultimaker Cura 2.6.2 using a layer height of 0.1 mm and the default printing parameters for thermoplastic polyurethane (TPU), namely Ultimaker TPU (white). The samples were printed on an Ultimaker 3+ using Ultimaker TPU (white). To experimentally map the stability design space (Figure S1c), the printed samples were manually inverted while the edges were kept clamped using a 3D printed test rig. The stability of the dome was categorized after an observation time of 1 minute. The sample is labelled monostable if it returns to the initial configuration immediately, bistable if it remains in the inverted configuration, and metastable if it returns to its base state not immediately, but after noticeable viscoelastic creep and relaxation in the inverted state.

Single dome characterization – Finite element analysis (FEA)

Using Abaqus/CAE 6.14 and Python 3.5 scripting, parametric models of the domes were created. The simulations were solved with Abaqus/Standard (implicit formulation). A linear-elastic material formulation with an elastic modulus of 26 MPa was used, according to the datasheet of the Ultimaker TPU filament (white). To account for the snap-through, a global damping value of 0.0001 was defined, which also enables potential metastability in the FEA models despite the idealized elastic material formulation. All edges were clamped during inversion and released before relaxation.

Abaqus data for domes of height 4 mm and thicknesses from 0.5 to 1.2 mm in 0.025 mm increments were imported to MATLAB R2015A, and a stability map for varying heights and thicknesses was generated (Figure S1c).

Curvature evaluation

A dome-patterned 1D strip design was employed for numerical characterization of the global curvature with respect to the local dome (unit cell) parameters. The dome-patterned strips consisted of 10 domes connected in series (1 × 10 pattern). An exhaustive parametric analysis was conducted for domes with base diameter 16 mm and with the dome and sheet thicknesses varying from 0.6 mm to 1 mm and dome heights varying from 4 mm to 6.5 mm. This allowed us to cover the bistability regime for the corresponding thickness values. The strips were modelled in Abaqus/CAE 6.14, and simulations were solved with Abaqus/Standard (implicit formulation). The material formulation was chosen analogous to the single dome FEA, with global damping values of 10^{-6} for the dome snap-through step and 10^{-8} for the subsequent relaxation steps. The simulations proceeded by first inverting the necessary domes using a displacement-controlled loading at the centers of the domes and constraining the corresponding internal and external edges of the strip. The loading and boundary conditions were subsequently released in an incremental manner for consecutive domes, allowing the strip to gradually relax to the final configuration. After relaxation, the data for one of the external edges of the dome sheet was extracted and post-processed in MATLAB R2016B to calculate the average global curvature using a circle fit algorithm based on the Taubin Method.[1] Upon repeated analyses, it was found that inverting either 5 or 6 domes (depending on the thickness and height values) in the 1D strips resulted in an arc shape with more than 180° of central angle. This in turn was enough to calculate the curvature value using the circle fit algorithm in a robust manner, which was found to remain constant as additional domes in the strip were inverted. The response surface over the dome height and thickness parameter space (Figure S2) was obtained using a linear interpolation fit to the data points.

Patterned metasheets for experimental characterization

The patterned sheets were created in analogy to the single domes using a parametric NX 10.0 model file and SolidWorks 2016 model files. Slicing was performed on Cura 3.4.0 and printing was carried out on an Ultimaker 3 and Ultimaker 3+ with Ultimaker TPU (white).

Finite element analysis of hierarchical sheets

The energy landscape of hierarchical sheets was calculated by finite element analysis using a full 3 × 3 dome pattern (Figure 2f, main text). The sheet was modelled in Abaqus/CAE 6.14, and the numerical analysis was run with Abaqus/Explicit. The chosen material formulation is analogous to that used for the FEA of the single dome, but with added material damping required for the explicit simulation. In the first step, all domes were inverted by applying corresponding loads onto the dome centers and boundary conditions between the dome regions. Subsequently, the boundary conditions were relieved, allowing the sheet to attain one of its globally stable states. From this stable state, small loads were applied at selected dome control points. The location and orientation of these small forces were chosen to favor of one of the alternative stable shapes. After the global snap-through was complete, the loads were removed, letting the structure relax in the newly attained shape. This procedure was repeated for each stable state. The total strain energy in the model was extracted to establish local energy minima of each globally stable shape (Figure 2f, main text)

Finite element analysis of localized pre-stressed areas

To numerically study the pre-stresses introduced upon dome inversion, we first evaluated an individual dome with pinned edges and subsequently analyzed a sheet featuring a 3 × 3 square dome pattern. The modelling was conducted in Abaqus/CAE 6.14 and simulations were solved with Abaqus/Standard (implicit formulation). The design parameters were dome height $h = 6$ mm, dome base radius $r = 8$ mm, thickness $t = 0.6$ mm and the side length of the square flat region surrounding each dome, which was fixed at 22 mm. The material formulation was chosen to be analogous to that used for the FEA of the single dome, with global damping

values of 10^{-6} for the dome snap-through step and 10^{-8} for the subsequent relaxation steps. The simulations proceeded by first inverting the necessary domes using a displacement-load at the center of the domes and constraining the corresponding internal and external edges of the sheet. The loading and boundary conditions were released in subsequent steps allowing the sheet to relax to the final configuration (Figure 3b, main text and Figure S6).

Bending and tensile tests on programmable sheets

To demonstrate the ability to program the global mechanical properties of the metamaterial sheet through local dome inversion, a design was selected that allows for the introduction of pre-stressed areas without changing the global shape of the sheet. The design consisted of a symmetrical layout of “up”- and “down”- facing domes in a checkerboard pattern. The resulting equal distribution of positive and negative curvatures introduced during dome inversion eventually leads to sheets with zero global curvature. The dome parameters were t = 0.8 mm, h = 5 mm, and r = 8 mm. The spacing was 20 mm × 20 mm and the strip contained 2×10 elements, leading to outer dimensions of 200 mm × 40 mm. Such sheets were generated and printed following the same procedure used to create single domes. The bending and tensile tests were conducted on a Shimadzu AGS-X using a 100N load transducer. The tensile tests were performed by applying a displacement rate of 20 mm/min up to a maximum load of 20 N. This load corresponds to 0.625 MPa in a flat, non-structured sheet with cross-sectional area 40 mm × 0.8 mm. The bending test was performed using large cylinders (d = 60 mm) as supports in order to minimize local interactions between the domes and the support. Both tensile and bending tests were run repeatedly in both base-state and fully-inverted sheets to prevent any effect of damage or other permanent change on the final measurement.

Manufacturing and testing of soft robotic gripper

The soft robotic gripper was designed in Siemens NX 10.0, sliced in Cura 3.4.0, and printed on an Ultimaker 3 using Ultimaker TPU (white). During the experiments, the gripper was clamped at its pressure inlet only. A special three-way connector was made to allow quick

switching between positive and negative pressures for the demonstrated two-way actuation of the domes. At each point of evaluation, the positive applied pressure values were read from the line gauge, whereas the negative ones were gathered from the vacuum pump. The demonstration video of the soft robotic gripper is uncut and runs in real time.

Manufacturing of morphologic sheets

The morphologic sheet was created using SolidWorks 2016 model files. Slicing was performed on Cura 4.3.0 and printing was carried out on an Ultimaker 3+ with Ultimaker TPU 95A (white).

Finite Element Analysis of morphologic sheets

The morphologic sheet was modelled in Abaqus/CAE 2018 and simulations were solved with Abaqus/Standard (implicit formulation). The material formulation was chosen analogous to the single dome FEA, with low global damping values ranging from 10^{-6} and 10^{-8} for the simulation steps. The simulations proceeded by sequentially inverting the domes using a displacement-load at the center of the domes and constraining the internal and external edges of the sheet. The boundary conditions were released and the morphologic sheet was allowed to relax after inversion of each dome set, thus allowing the sheet to relax sequentially to the desired global configuration based on the order in which the domes are inverted. The morphologic sheet is designed using domes with dimensions' $r = 8$ mm, $t = 0.6$ mm, $h = 4$ mm and spacing 22 mm. The connecting beam-like members have length 30 mm, width 4 mm and thickness 0.6 mm and the vertical output node has dimensions' length 66 mm, width 2 mm and thickness 0.6 mm.

Manufacturing of Nylon metamaterial sheet

The metamaterial sheet was created using SolidWorks 2016 model files. Slicing was performed on Cura 4.3.0, and printing was carried out on an Ultimaker 3+ with Ultimaker Nylon (transparent).

Finite Element Analysis of tensile loading tests on PLA and TPU metamaterial sheets

The sheets were modeled in Abaqus/CAE 2018, and simulations were solved with Abaqus/Standard (implicit formulation) with nonlinear geometric effects. A linear-elastic material formulation was used for both polylactic acid (PLA; modulus 2346.5 MPa, Poisson's ratio 0.3) and TPU (modulus 26 MPa, Poisson's ratio 0.3) according to the Ultimaker materials datasheet. Global damping value of 10^{-6} was employed for the dome snap-through step and 10^{-8} for the subsequent relaxation steps. The simulations proceeded by first inverting the necessary domes using displacement-controlled loading at the centers of the domes and constraining the corresponding internal and external edges of the strip. The loading and boundary conditions were subsequently released in an incremental manner for consecutive domes, allowing the sheet to gradually relax to the final configuration. After relaxation, a tensile displacement load was applied in a final loading step (damping value 10^{-6}) at one end, while keeping the other end constrained. The reaction-force and displacement data at the loaded end were then extracted and post-processed in MATLAB R2016B to obtain the tensile force-displacement curves.

2. Mechanics model

Correlating the global curvature of our metamaterial sheets with the local deformation arising from the inversion of individual domes requires an understanding of the mechanics of single domes and their effect on the collective deformation of multiple domes. The mechanics and inversion of domes have been extensively studied.[2-5] The behavior of such doubly curved shells is generally characterized by the shell geometry, principally the dome height and thickness, and by the Poisson's ratio of the constituent material. However, the effect of elastically constraining such spherical shells to elastic structures has been less explored, with only a few studies considering effects of lumped elastic connections on the mechanics of spherical shells.[6]

The bistable behavior of individual domes was studied with the help of simulations and experiments (Figure S1). The bistability phenomenon is associated with the presence of two potential wells in the energy landscape of the system (Figure S1d). The energy barrier that separates these wells arises from the elastic strain energy stored in the dome as it is forced into a flattened geometry between the two curved states. FE simulations were carried out to determine the geometrical parameters required for the appearance of the two potential wells in the energy landscape. The minimum dome height estimated from the simulations shows close agreement with the experimental data (Figure S1c).

Patterning the individual unit cells into 1D metastrip designs (Figures 2a-2c, main text), it is observed that local inversion of individual domes leads to global changes in the metamaterial shape, resulting in a transformation from a flat 1D strip to a curved surface featuring out-of-plane deflection. The curvature of the global metamaterial configuration can be tuned by varying the geometry of the individual domes. Figure S2 illustrates a 3D map depicting the effect of individual dome height and thickness on the effective global curvature (2D version of this plot is shown in Figure 2c in the main manuscript). The results indicate that the global strip curvature depends linearly on the dome height and is, for the most part, independent of the thickness. Both of these trends hold true for sufficiently small dome heights (h < 6 mm). A deviation from these trends is observed as the dome height approaches the value of the base radius (hemispherical designs).

To understand the dependence of the global curvature on the local geometry of the domes, we propose a mechanical model that considers the energetics of the metamaterial sheet. The flat area surrounding the domes in our metamaterial sheets imposes continuous elastic constraints on the shell boundaries. Analyzing the mechanical response of several of these connected units resorting to the use of partial differential equations would constitute a difficult boundary value problem. Instead, we utilize an energetics approach to investigate the relationship between the unit cell (dome) local geometry and the global deflection of dome strips, which in turn, is characterized by its global

curvature. In particular, we quantify the energy associated with the pre-stress introduced by the local inversion of domes and correlate it to the attained global curvature of a strip (Figure S3a).

In the zero-stress state, the strip is in its globally flat, undeformed configuration while in the pre-stressed or the inverted state, the strip assumes a curved global configuration. For the following analysis, we assume shallow shell theory remains valid for the range of dome geometries and Poisson's ratios investigated. Following the analysis by Seffen and Vidoli,[4] the energy required for inverting a single unconstrained dome, u_b, is proportional to $\frac{Et^3}{(1-\mu^2)}\frac{4h^2}{R^2}$, where E, μ, h, t and R are the elastic modulus, Poisson's ratio, thickness, height and radius of the domes respectively. Furthermore, since the stretching energy associated with the inversion of thin domes is minimal ($u_s \ll u_b$), one can assume that the work done for inverting a free dome W_{in}^{f} is approximately equal to the bending energy $W_{in}^{f} \approx u_b$. This assumption was confirmed by FE calculations of the energy required for inversion as a function of the dome height.

FE simulations on a dome-patterned strip indicate that the work required to invert an identical dome on a strip W_{in}^{c} is greater than the work W_{in}^{f} associated with free dome inversion, such that $W_{in}^{c} = \alpha W_{in}^{f}$ (Figure S3b-d). We hypothesize that this work differential, $W_{in}^{c} - W_{in}^{f}$, is converted into global bending of the strip. FE analysis of the strip confirms that the global strain energy increases by a constant value ΔE as an increasing number of identical domes are sequentially inverted (Figure S3b). These observations together suggest that a constant amount of energy contributes to global bending of the strip upon each successive dome inversion (Figure S3c). The total amount of energy can then be expressed as $W_b = \sum_1^N n(W_{in}^{c} - W_{in}^{f}) = \sum_1^N n(\alpha - 1)W_{in}^{f}$, where n is the number of domes inverted and the proportionality constant, α, accounts for the correction term in the inversion energy. This expression leads to the simple relationship: $W_b \propto \frac{Et^3}{(1-\mu^2)}\frac{4h^2}{R^2}$. For the strip, we assume an inextensible homogenized beam model with global bending energy: $U_b = \frac{1}{2}E(b)_{eq}t^3\kappa^2 L = W_b$, where $(b)_{eq}$ and κ are the equivalent width and global curvature of the strip, respectively.

Therefore, the global curvature of the strip, κ, is proportional to $\left[\frac{\frac{Et^3}{(1-\mu^2)}\frac{4h^2}{R^2}}{\frac{1}{2}E(b)_{eq}t^3L}\right]^{\frac{1}{2}}$. This implies that the global curvature is linearly dependent on the height of the unit cell domes, $\kappa \propto h$, and independent of the thickness of the strip. Our energetics analysis matches closely to the FE results, thus validating our hypothesis that the differential work between the constrained and unconstrained unit cells is converted into global bending of dome strips (Figure 2a-c, main text and Figure S2).

3. Surface family selection from multistable metasheets

A TPU sheet comprising a square array of 8 × 8 domes was fabricated using an Ultimaker 3+ fused deposition modelling (FDM) printer (Figure S4). All the domes in the metamaterial sheet have uniform dimensions with height h = 4.5 mm, and base radius r = 8 mm. The sheet is printed with thickness t = 0.8 mm and has overall length and width of 160 mm, with uniform spacing of 20 mm between the centers of neighboring domes. Each individual dome features two stable configurations: the as-printed base state and the inverted state, both of which are illustrated in Figure S1b. Theoretically, these two possible local states would result in a total of 2^N stable global configurations. However, the emergence of hierarchical multistability at the global scale leads to an explosion of stable states beyond the possible local configurations. A remarkable level of shape complexity and multiple stable states are achievable with these hierarchical metamaterials, some of which are illustrated in Figure 3a in the main manuscript. Cylinders, star traps and saddles can be generated, depending on the pattern of local inversions of the domes (unit cells). However, shapes are not limited to these arbitrary classifications. When individual domes are inverted in a non-centrosymmetric fashion, a large variety of complex, and in some cases non-symmetric, hierarchical multistable states can be obtained (Figure S5).

4. Localized areas of pre-stress for property tunability

Inversion of a bistable dome results in strain energy concentration along the circumference of the dome, as illustrated by simulations of an individual dome with pinned edges (Figure S6a). This local

pre-stress effect can be leveraged for tuning the global mechanical behavior of the metamaterial sheet. Inversion of domes in a 2D metamaterial sheet allows for the introduction of localized areas of pre-stress in the sheet. Pre-stressed locations can be programmed and manipulated by selectively inverting different and/or additional domes in the metamaterial sheet. This feature is exemplified with the help of a 3 × 3 metamaterial sheet in Figure S6 and in Figure 3b of the main manuscript. Figure S6 illustrates the ability to introduce localized areas of pre-stress by inverting 1 or 3 domes in an otherwise stress-free reference state. The possibility to program the global mechanical behavior of the metamaterial sheet through the introduction of these pre-stressed areas is evidenced by the tunable force-displacement relations for sheets subjected to bending and tensile loading (Figures 3c and 3d main manuscript).

5. Muscle-free pneumatic robotics with programmed minimum-energy states

Exploiting the intrinsic reconfiguration afforded by our dome-patterned metasheets allows for designing soft robots. The infinite-dimensional configuration space of deformations is constrained by the presence of domes, enforcing a known number of discrete positions. Importantly, the discrete stable states of such a robot can be accessed in response to a single input and without close-loop control by exploiting the minimum-energy property imposed by our multistable metasheets. Open-loop pressurization without precise control of the input magnitude still leads to the same final programmed state as the system reaches an encoded minimum-energy equilibrium (Movie S3). Material viscoelastic effects are not found to affect the final shape, and only a minor delay in the time needed to attain the final configuration is observed. Accessing different possible configurations can be attained by programming domes with increasing heights which require different pressure inputs to be inverted (Movie S2). Significantly, these programmed global shapes can be accessed with a single control input (pressure level) and no feedback, thus providing a general method for simplifying the sensing and actuation control problems currently impeding the progress of soft robotics.[7]

6. Morphologic computation with multistable metasheets

The spatial distribution of local dome inversions and more importantly, the dome inversion history leading to a given final pattern is reflected in the attained global hierarchical shape of our metamaterial sheets (Figure S7). This concept is illustrated by selecting the dome inversion history to achieve a final left-twisting shape (Figure S7a and Movie S5), right-twisting shape (Figure S7b and Movie S6) and cylindrical shape (Figure S7c, Movie S7), all of which co-exist at the global scale for a given pattern of dome inversion. This intrinsic dependence of a hierarchical shape on the dome inversion history can be leveraged to encode path-dependent logic into the metamaterial architecture. Local inversion of individual unit cells enables memory storage in binary form, with the ground state as 0 and inverted state as 1. More importantly, the history dependence of the global shape encodes temporal memory of the inputs in the final hierarchical state adopted by our dome-patterned metamaterial. In essence, the local unit cell states store information, whereas the final shape stores the history and spatial pattern of inversion. Thus, our morphologic metamaterial extends mechanical computation from the current transistor-based logic to a more complex paradigm that couples sensing and in-memory operations.

A morphologic metamaterial sheet featuring domes in a 3 × 3 square pattern (Figure S8a) is investigated as a prototype for conducting mechanical computations. When all domes are inverted, the metasheet exhibits hierarchical multistability and the co-existing global shapes fall under 2 distinct families – bending-dominated shapes (Figure S8b) and twisting-dominated shapes (Figure S8c). Each family of shapes is associated with a different energy level (Figure S8d). A desired shape family or sub-shapes within a given shape family featuring different orientations can be accessed by employing different dome inversion histories. Thus, the final shape of the 3 × 3 metasheet with all domes inverted encodes the spatiotemporal distribution of the mechanical input (Figure S8d). This characteristic is leveraged to design a morphologic substrate featuring two 3 × 3 metasheets physically connected together by beam-like links. Each metasheet corresponds to an input node of the morphologic substrate and the thin vertical line constitutes the observable output node (Figures

S8e). Drawing analogies to classical state-logic conventions, input fields resulting in dome inversion histories leading to the bending shape family are ascribed a value of 0 and input fields with dome inversion histories leading to the family of twisting shapes are ascribed a value of 1. Based on the shape of each of the input nodes, the output node yields the deformation compatibility between the respective nodes. In this regard, a straight line configuration of the output node is assigned the value 0 and a rotated, slanted line configuration is assigned the value 1 (Figures S8f).

The architecture of the proposed morphologic substrate can be represented in an abstract logic diagram (Figure 4f main manuscript). The combined gate or input nodes are indicated by CG1 and CG2 (grey blocks), whereas the output node is indicated by O1 (blue block). The nodes T1 and T2 (brown triangles) refer to the transduction elements that may be incorporated within the domes of our morphologic substrate to make them responsive to various input fields, such as temperature, pressure, humidity, electrical and magnetic fields or other environmental stimuli (indicated by A and B). Communication channels (solid lines) carry both internal and external (C and D) information that is processed through the morphologic substrate to yield an output (O1). In our dome-patterned metasheet, such information comprises the spatial distribution of the inverted domes, the order/sequencing of dome inversion and the global deformation of each of the combined gate nodes. Cross-feedback channels between the combined gate nodes (dashed lines) capture the interaction between the two physically-linked metasheets. The strength of the interaction can be altered by the design of the beam-like connections between the combined gate nodes. Finally, the output node O1 can lead to an observable integrated decision as readout (E). This output can be tailored to allow for unique readouts relating to sensing or computation in reconfigurable structures, such as soft robots or morphing structures.

To illustrate some of the morphologic operations that can be executed with this particular design of our morphologic substrate, we put together a state-transition table that depicts the output line expected for different possible inputs (Figure S9). When both of the combined gates of the morphologic substrate are subjected to inversion sequences with compatible twisting (Input1:CG1 =

1 and Input2:CG2 = 1), the mechanical output node becomes slanted, thereby indicating an output value, O1 = 1 (Figure 4d.i main manuscript and Movie S8). On the contrary, if the combined gates of the morphologic substrate are activated via inputs leading to shapes belonging to the bending family (Input1:CG1 = 0 and Input2:CG2 = 0), the mechanical output readout node remains straight (O1 = 0; Figure 4d.ii main manuscript and Movie S9). Additionally, if the input fields on combined gates lead to a bending shape on one gate but a twisted shape on the other gate (Input1:CG1 = 1 and Input2:CG2 = 0), the mechanical output node remains straight (O1 = 0; Figure 4d.iii main manuscript and Movie S10). In essence, the state transition table of this design of our morphological substrate is similar to an AND logic gate. However, unique to our morphological substrate is the ability to resolve physical input fields with complex spatiotemporal distributions to yield the presented simplified logic output.

7. Role of constituent material modulus on hierarchical multistability and property programmability

A Nylon sheet featuring a square array of 3 × 3 domes was fabricated using an Ultimaker 3+ fused deposition modeling (FDM) printer (Figure S10a). Nylon has an elastic modulus of 579 MPa, which is 22 times greater than thermoplastic polyurethane. With a glass transition temperature above 50°C, Nylon shows no viscoelastic effect at room temperature. All domes in the Nylon-made metamaterial sheet have uniform dimensions with height h = 4 mm, base radius r = 12 mm, thickness t = 0.4 mm and uniform spacing of 28 mm between centers of neighboring domes. Locally, each individual dome features two stable configurations: the as-printed base state and the inverted state. At the global scale, the nylon sheet is again found to exhibit hierarchical multistability with the emergence of the twisting state (Figure S10b) and the bending state (Figure S10c) when all the domes are inverted. This result indicates that hierarchical multistability is not a result of viscoelastic effects in the constituent material and can be realized in dome metamaterial sheets that are made of constituent materials featuring a high elastic modulus as well. Building on these experimental observations, FE simulations were carried out to compare the force-displacement response of metamaterials sheets designed

using Polylactic Acid (PLA, modulus 2346.5 MPa, Poisson's ratio 0.3) and Thermoplastic Polyurethane (TPU, modulus 26 MPa, Poisson's ratio 0.3) as the constituent materials. The results for metamaterial sheets featuring the same design indicate that structures made of PLA can carry significantly more load compared to those featuring TPU as the constituent material (Figure S11). The metamaterial sheets display a 2 × 10 checkerboard pattern with dome dimensions, h = 3 mm, r = 12 mm, t = 0.2 mm and uniform spacing of 33.6 mm between centers of neighboring domes. It is noted that the maximum in-plane principal logarithmic strain in all the domes in the inverted state and after application of the tensile displacement load is less than 2×10^{-2} i.e. under 2%. This suggests the metamaterial sheet can be designed to achieve higher modulus, that as long as the constituent material can sustain 2% strains. In turn, this increases the load carrying capability of the metamaterial sheet, thus opening the door for using a variety of hybrid composites for manufacturing the dome metamaterials.[8]

8. Figures S1-S9

Supplementary Figure S1

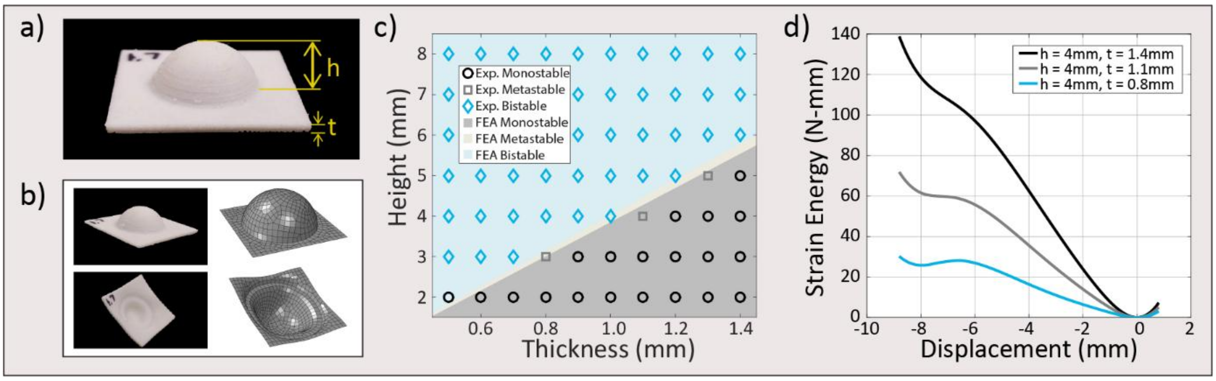

Figure S1: Geometry and mechanics of an individual soft dome during inversion. (a) Relevant geometrical parameters of an individual 3D printed dome. The dome height and the sheet thickness are given by *h* and *t*, respectively. (b) Shape change induced by the inversion of a 3D printed (left) and a simulated (right) dome. (c) Geometrical conditions necessary to achieve dome bistability. Background colors indicate the simulation results, whereas the symbols show experimental data. (d) Monostable (black), metastable (grey), and multistable (blue) energy landscape, depending on the dome aspect ratio.

Supplementary Figure S2

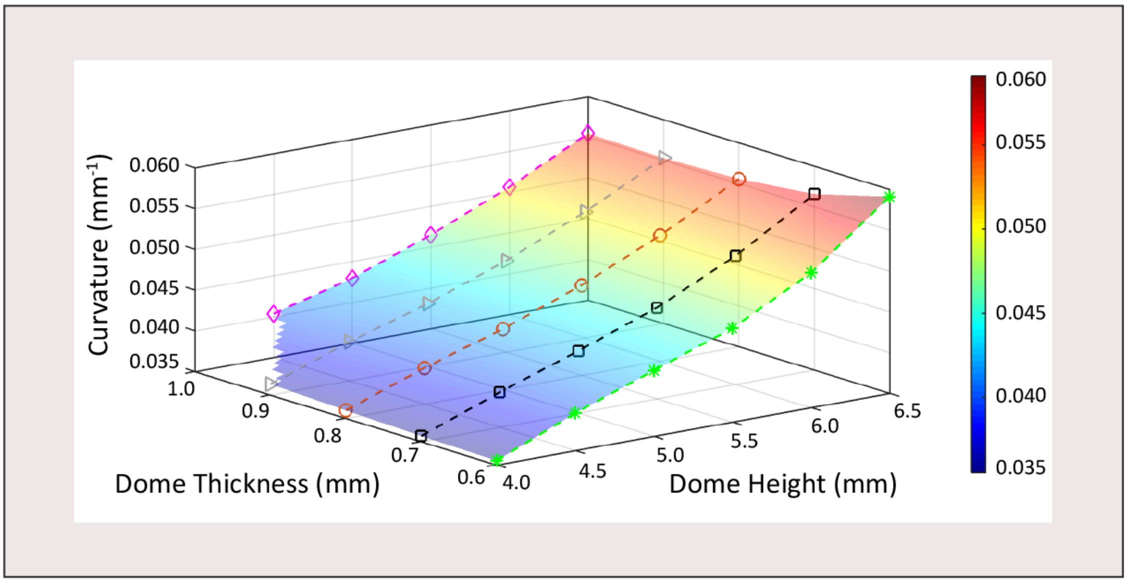

Figure S2: Results of the Finite Element simulations illustrating the effect of dome height and thickness on the global curvature of 1D strips after inversion of domes.

Supplementary Figure S3

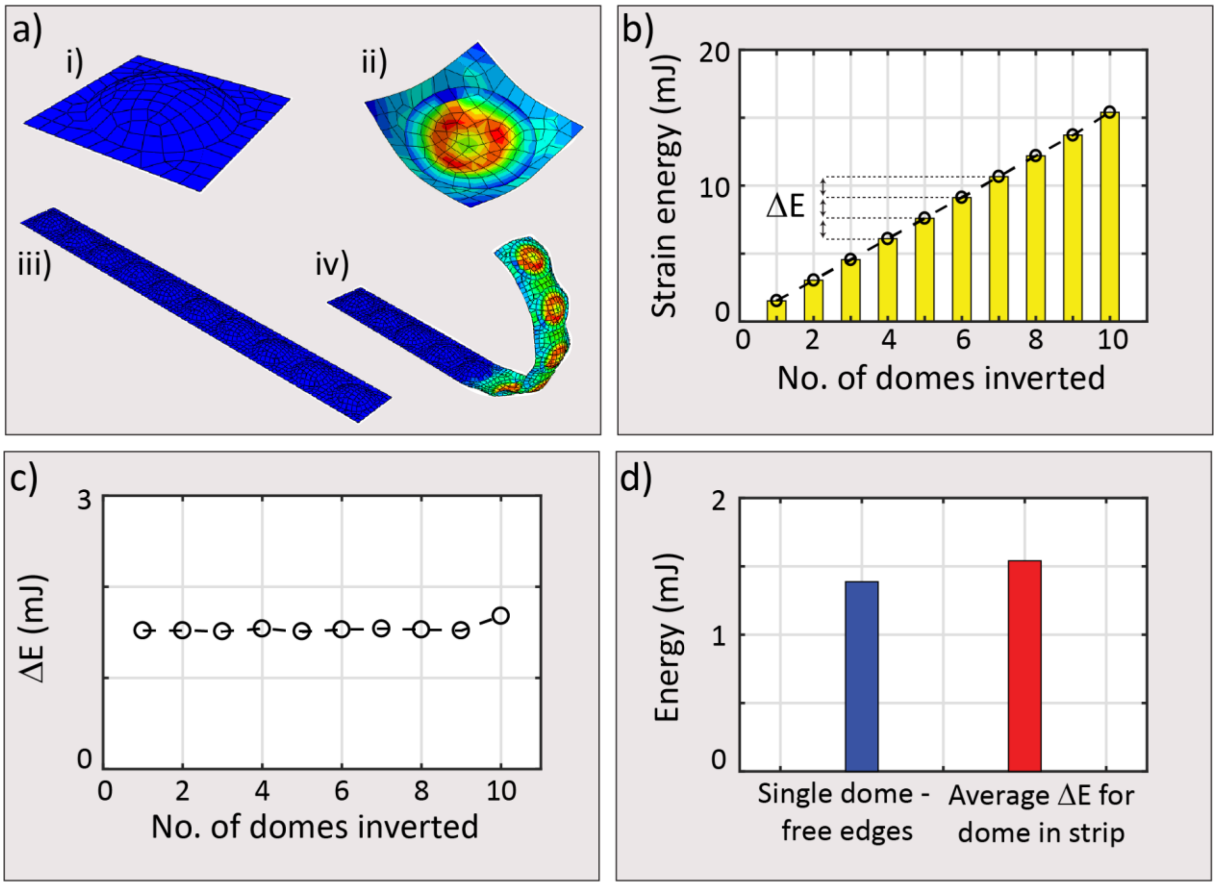

Figure S3: Finite element simulations of individual domes and dome-patterned strips. (a) Single unit comprised of a dome and the surrounding flat area in its (i) undeformed and (ii) inverted configurations. As single domes are inverted the dome-patterned strip deforms from its (iii) globally flat to (iv) curved configurations. (b) Work added onto a 10-dome strip by successive inversion of individual units. (c) The energy differential ΔE between successive inversions remains constant, regardless of the number of inverted domes. (d) The energy of inverting successive units on a strip is larger than the energy required to invert a stand-alone unconstrained unit.

Supplementary Figure S4

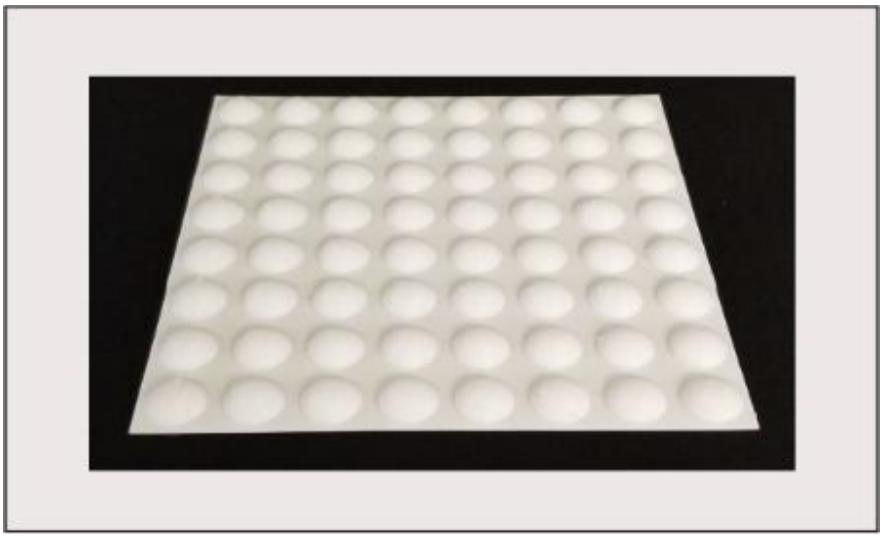

Figure S4: 3D printed thermoplastic polyurethane sheet displaying an array of 8 × 8 square domes in the as-printed base state (no domes inverted).

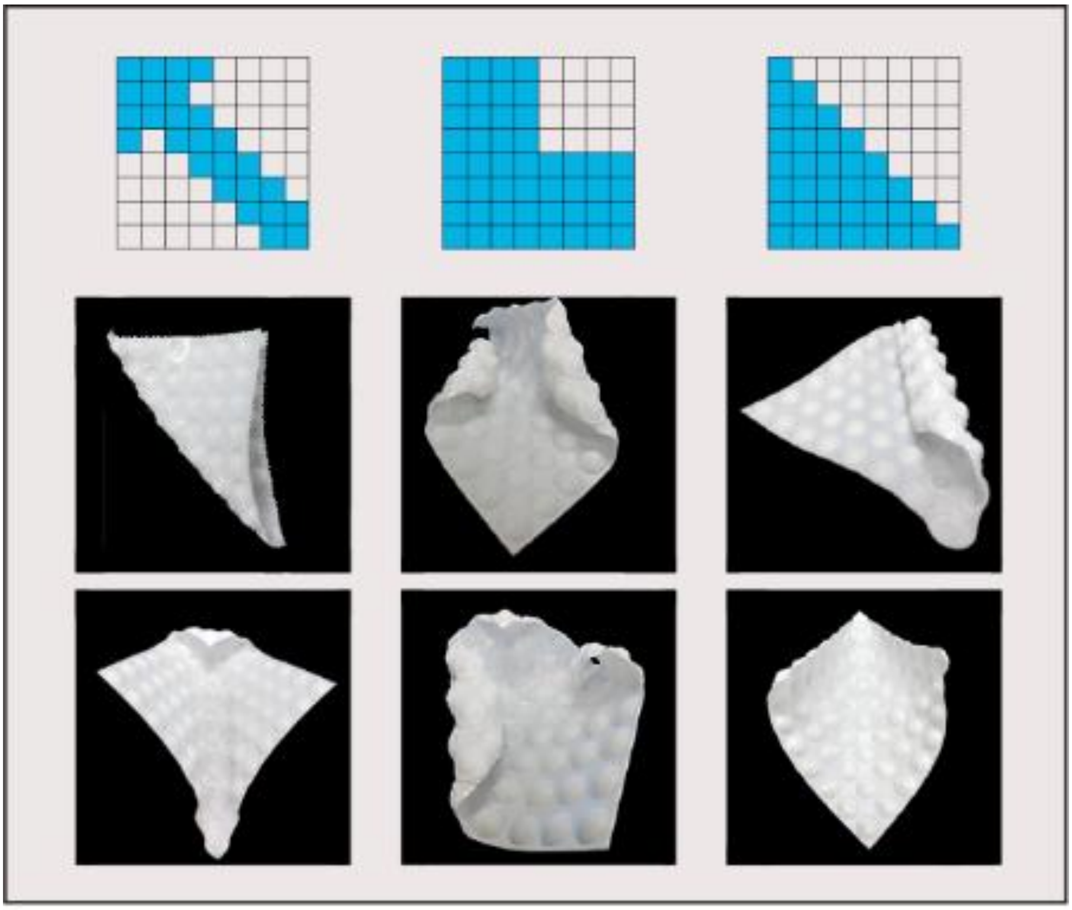

Supplementary Figure S5

Figure S5: Examples of complex multistable global shapes that can be generated using 8 × 8 sheets with non-centrosymmetric patterns of inverted domes.

Supplementary Figure S6

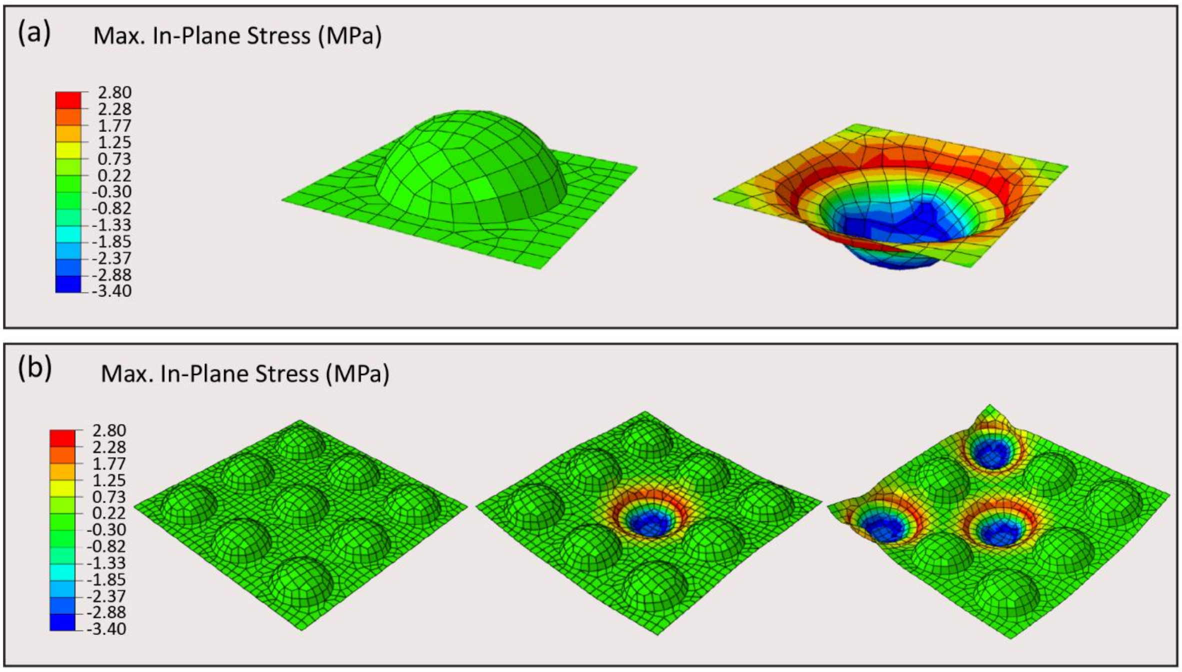

Figure S6: (a) Finite Element analysis of a single dome with pinned edges showing the stresses developed upon inversion. (b) Finite Element analysis of a 3 × 3 sheet with 0 domes, 1 dome and 3 domes inverted, left to right. This simulation illustrates that the localized areas of pre-stress resulting from the inversion of an individual dome are not significantly affected by the state of adjacent domes in the sheet. Design parameters are dome height h = 6 mm, dome base radius r = 8 mm, thickness t = 0.6 mm and the side length of the square flat region surrounding each dome, which is fixed at 22 mm in this example.

Supplementary Figure S7

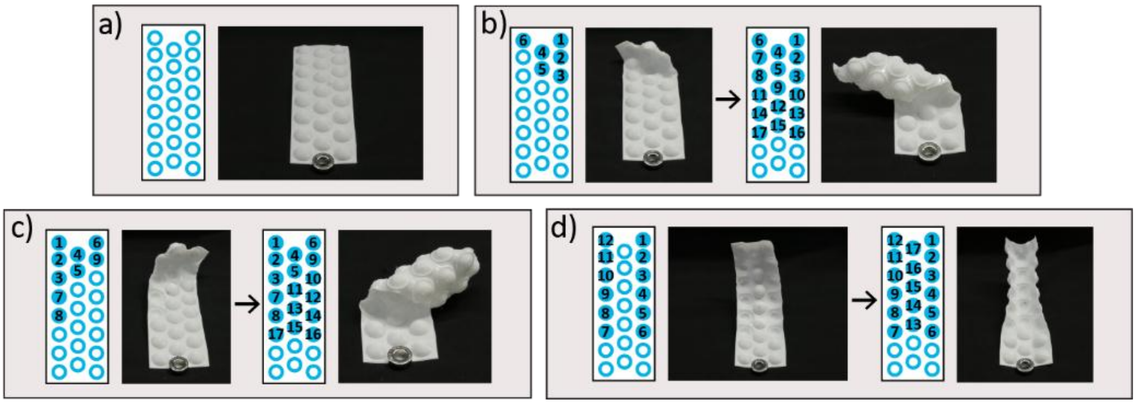

Figure S7: Memory capability of the morphologic metamaterial sheets. Examples of the local dome inversion history leading to a particular desired global shape amongst other coexisting stable hierarchical shapes, three of which are shown in Movies S5-S7. (a) Zero stress state. (b-d) Three different dome inversion histories and associated intermediate shapes leading to the same final inversion pattern, yet reaching three different stable hierarchical shapes: (b) twist-left (Movie S5), (c) twist-right (Movie S6), (d) cylindrical shape (Movie S7). The numbers indicate the sequence of dome inversion.

Supplementary Figure S8

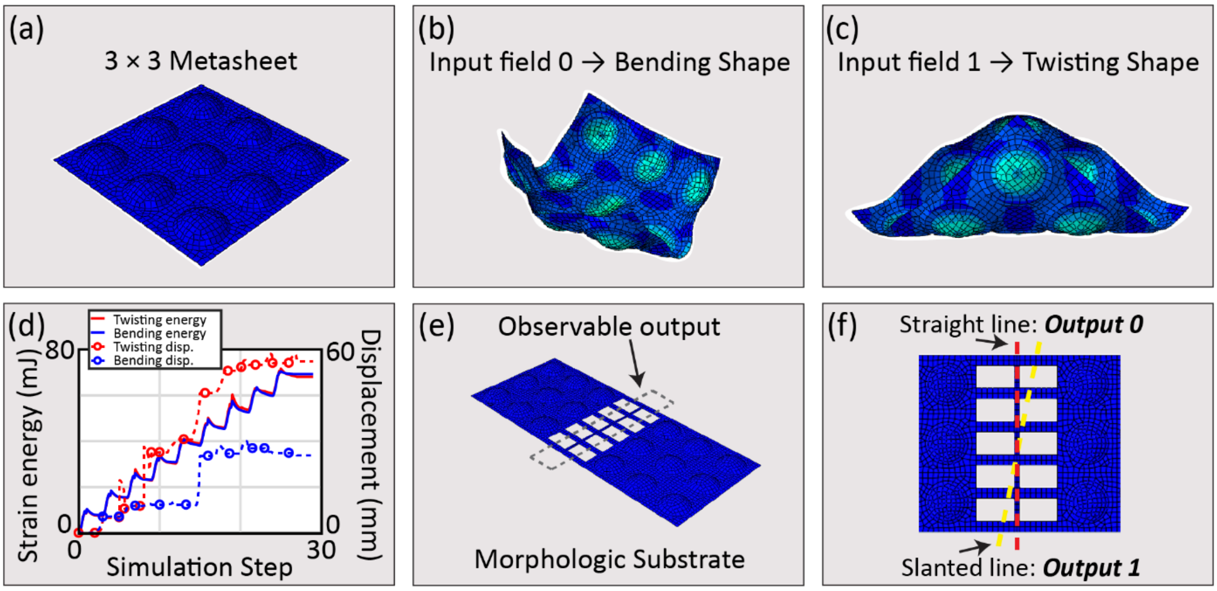

Figure S8: Logic operation using the morphologic metamaterial sheets. (a) 3 × 3 metasheet as combined gate for spatiotemporal mechanical computation. Inputs leading to (b) global bending and (c) twisting states are assigned values 0 and 1 as inputs to the sheet as each shape is reached by a specific inversion pattern sequence, thus encoding spatiotemporal information about the deformation input. (d) FE simulation of two different dome inversion patterns (inset blue and red) that lead to the two global states (b) and (c), respectively. Simulations are conducted by inverting a different sequence of domes leading to the same final pattern. However, due to the variation in the sequence, the final strain energy value and the final displacement magnitude value of a representative (the corner node) is different, indicating that a different global minimum is reached. (e) Morphologic substrate made by physically connecting two metasheets i.e. combined gate units by an observable mechanical output. (f) The deformation of the output line indicates 1 if slanted (dashed yellow line) or 0 if straight (dashed red line).

Supplementary Figure S9

Morphologic Substrate

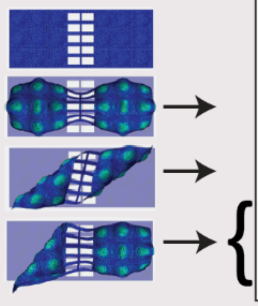

State Transition Table

Input 1: CG1	Input 2: CG2	Output: O1
0	0	0
1	1	1
0	1	0
1	0	0

Figure S9: State-transition table and simulated associated global shapes adopted by morphologic substrate. The global shape of the connected hierarchical multistable sheets reflects the spatiotemporal input provided to each combined gate (left and right sheets). Global shapes leading to a slanted output line is encoded as 1, whereas 0 corresponds to a straight output line.

Supplementary Figure S10

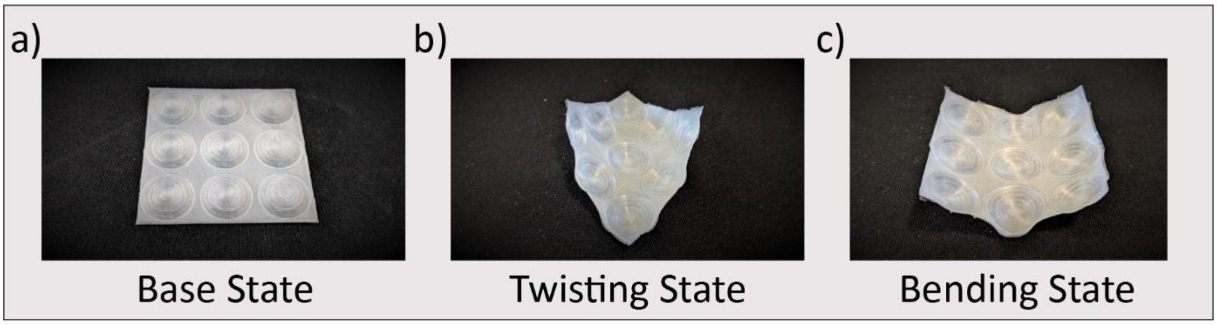

Figure S10: 3D printed Nylon sheet displaying a square array of 3 × 3 square domes in the (a) as-printed base state (no domes inverted), (b) twisting state with all domes inverted and, (c) bending state with the domes inverted.

Supplementary Figure S11

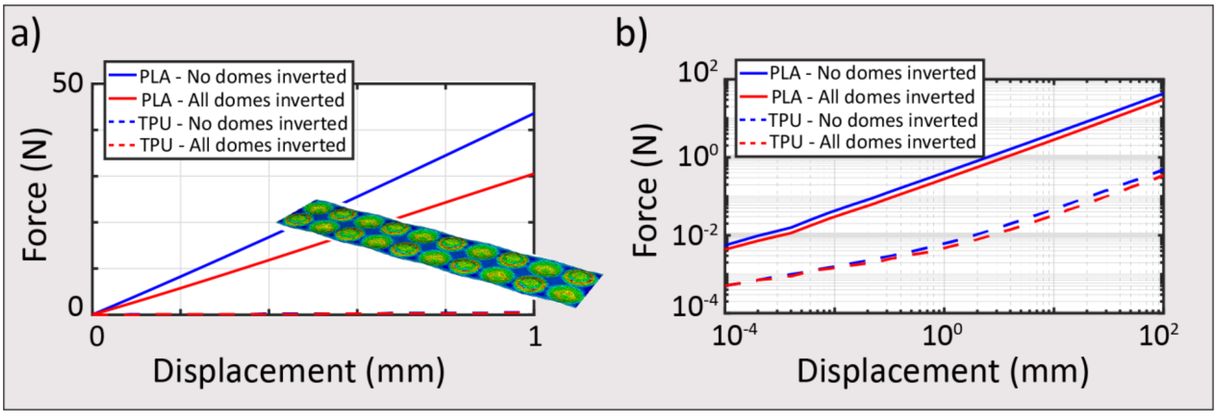

Figure S11: FE simulations comparing the tensile load-displacement response in the (a) linear scale and (b) logarithmic scale, of a 2 × 10 metamaterial sheet featuring square domes facing up and down in a checkerboard pattern designed using PLA and TPU as the constituent materials.

9. List of Supplementary Videos:

1. Movie S1: Programmable gripper. The inversion of local domes adapts the global shape, closing the grip, while the stored strain energy allows for holding onto an object without need for continuous external energy provision.
2. Movie S2: Experimental demonstration of geometrical design of domes to achieve inversion at specific pressure levels.
3. Movie S3: Repeatability of achieving the final state under different pneumatic pressure levels.
4. Movie S4: Final shape of metasheet is independent of the inversion order for small number of inverted domes.
5. Movie S5: Inversion history-dependent global shape for large number of inverted domes. The specific inversion pattern reaches one of the available coexisting global shapes: a left-handed helix.
6. Movie S6: Inversion history-dependent global shape for large number of inverted domes. The specific inversion pattern reaches one of the available coexisting global shapes: a right-handed helix.
7. Movie S7: Inversion history-dependent global shape for large number of inverted domes. The specific inversion pattern reaches one of the available coexisting global shapes: a cylindrical shell.
8. Movie S8: Simulation showing an input field inversion pattern resulting in collective twisting of both sheets and Output readout 1 on a morphologic substrate.
9. Movie S9: Simulation showing an input field inversion pattern resulting in bending of both sheets and Output readout 0 on a morphologic substrate.
10. Movie S10: Simulation showing an input field inversion pattern resulting in twisting of one sheet and bending of the other sheet with Output readout 0 on a morphologic substrate.

10. References

1. "Estimation of Planar Curves, Surfaces and Nonplanar Space Curves Defined by Implicit Equations, with Applications to Edge and Range Image Segmentation", IEEE Trans. PAMI, Vol. 13, pages 1115-1138, (1991)
2. Vaziri, A. & Mahadevan, L. Localized and extended deformations of elastic shells. *Proc. Natl. Acad. Sci.* **105**, 7913–7918 (2008).
3. Vaziri, A. Mechanics of highly deformed elastic shells. *Thin-Walled Struct.* **47**, 692–700 (2009).
4. Seffen, K. A. & Vidoli, S. Eversion of bistable shells under magnetic actuation: a model of nonlinear shapes. *Smart Mater. Struct.* **25**, 65010 (2016).
5. Pezzulla, M., Stoop, N., Steranka, M. P., Bade, A. J. & Holmes, D. P. Curvature-Induced Instabilities of Shells. *Phys. Rev. Lett.* **120**, 48002 (2018).
6. Sobota, P. M. & Seffen, K. A. Effects of boundary conditions on bistable behaviour in axisymmetrical shallow shells. *Proc. R. Soc. A Math. Phys. Eng. Sci.* **473**, 20170230 (2017).
7. Laschi, C. & Mazzolai, B. Lessons from Animals and Plants: The Symbiosis of Morphological Computation and Soft Robotics. *IEEE Robot. Autom. Mag.* 107–114 (2016) doi:10.1109/MRA.2016.2582726.
8. Czél, G. & Wisnom, M. R. Demonstration of pseudo-ductility in high performance glass/epoxy composites by hybridisation with thin-ply carbon prepreg. Compos. Part A Appl. Sci. Manuf. 52, 23–30 (2013).
